# Regulation of potassium dependent ATPase (*kdp*) operon of *Deinococcus radiodurans*

**DOI:** 10.1371/journal.pone.0188998

**Published:** 2017-12-05

**Authors:** Pratiksha Dani, Aman Kumar Ujaoney, Shree Kumar Apte, Bhakti Basu

**Affiliations:** 1 Molecular Biology Division, Bhabha Atomic Research Centre, Mumbai, India; 2 Homi Bhabha National Institute, Training School Complex, Anushakti Nagar, Mumbai, India; Niels Bohr Institute, DENMARK

## Abstract

The genome of *D*. *radiodurans* harbors genes for structural and regulatory proteins of Kdp ATPase, in an operon pattern, on Mega plasmid 1. Organization of its two-component regulatory genes is unique. Here we demonstrate that both, the structural as well as regulatory components of the *kdp* operon of *D*. *radiodurans* are expressed quickly as the cells experience potassium limitation but are not expressed upon increase in osmolarity. The cognate DNA binding response regulator (RR) effects the expression of *kdp* operon during potassium deficiency through specific interaction with the *kdp* promoter. Deletion of the gene encoding RR protein renders the mutant *D*. *radiodurans* (ΔRR) unable to express *kdp* operon under potassium limitation. The ΔRR *D*. *radiodurans* displays no growth defect when grown on rich media or when exposed to oxidative or heat stress but shows reduced growth following gamma irradiation. The study elucidates the functional and regulatory aspects of the novel *kdp* operon of this extremophile, for the first time.

## Introduction

Potassium (K^+^) is essential for physiological functions such as regulation of intracellular pH, transmembrane electrical potential and turgor pressure in all living organisms [1]. K^+^ homeostasis is critical for adaptation to several biotic or abiotic stresses in bacteria [1–6]. Bacteria accumulate 0.1–0.6 M K^+^ intracellularly from trace amounts (0.1–10 mM) of this cation present in the environment [7] through a variety of low and high affinity K^+^ transporters. In *E*. *coli*, low affinity K^+^ transporters Trk and Kup are constitutively expressed and maintain physiological concentration of potassium in the cell [8] while high affinity ATP-dependent K^+^ uptake system (K_m_ 2 μM), is effected by an inducible KdpATPase expressed under K^+^ limiting conditions (active below 5 mM K^+^) [5]. Ktr, a fourth constitutively expressed K^+^ uptake transporter, is absent in *E*. *coli* but encoded by several other microbes [9].

The homologs of *kdp* operon and its two-component signaling system are present in Gram-positive as well as Gram-negative bacteria and archaeal species [10] wherein *kdp* operon expression is stimulated by low K^+^ concentration and high osmolarity. In addition, its expression has also been coupled with regulation of virulence genes and pathogenesis in *Staphylococcus aureus*, *Salmonella typhimurium*, *Yersinia pestis*, mycobacteria etc. [11, 12] Expression of *kdp* operon is also associated with high NaCl tolerance in *S*. *aureus* [12], phosphate limitation in *E*. *coli* [13], or drought stress in *Anabaena* 7120 [14]. Thus, *kdp* operon forms a part of basic metabolism and also contributes to survival under a variety of stressful conditions.

Gram-positive *D*. *radiodurans* exhibits extreme resistance to gamma radiation as well as to desiccation [15], owing to its customized DNA damage repair system, enzymatic/non-enzymatic antioxidants and metabolites. A number of elegant studies reported differential expression/levels of transcriptome, small RNAs, proteome, antioxidants, metabolites etc. during the phase of DNA repair [16–21]. K^+^ is necessary for the stability of replication process and maintaining genome integrity in cells [22, 23], both the functions actively carried out by *D*. *radiodurans* while recovering from DNA damage. Thus, role of K^+^ homeostasis in the stress resistances of *D*. *radiodurans* needs detailed exploration.

**Fig 1:**
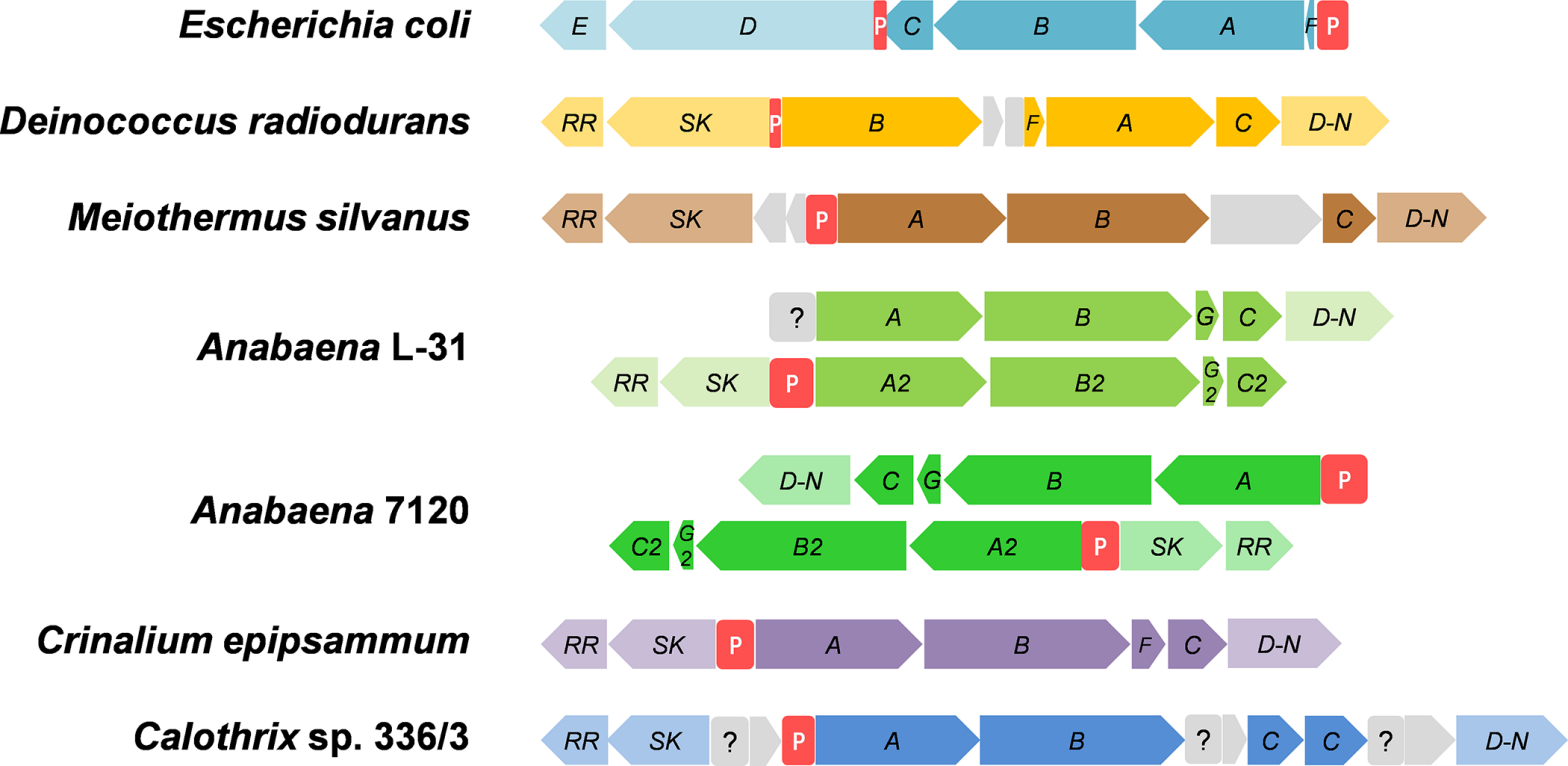
Comparison of organization of *kdp* operon in bacteria. Organization of *kdp* operon in *E*. *coli* (model organism), representative examples from *Deinococcus-Thermus* group and cyanobacteria (as depicted in KEGG database [29]). The promoter regions are shown with red boxes denoted by the letter ‘P’. Undefined promoter region is shown with a gray box and question mark. *A*, *B*, *C*, *D*, *E* and *F* denote *kdpA*, *kdpB*, *kdpC*, *kdpD*, *kdpE* and *kdpF* genes, respectively. *RR*, *SK* and *D-N* denote response regulator, sensor kinase and N-terminal half of *kdpD* gene, respectively.

The genome of *D*. *radiodurans* shows presence of two K^+^ uptake systems, namely, Ktr and K^+^-transporting ATPase (Kdp). The organization of *kdp* genes in *D*. *radiodurans* is distinct from well studied *kdp* operon in *E*. *coli*, especially in the two component regulatory genes (Fig 1). In *D*. *radiodurans*, structural genes *kdpBAC* (DR_B0083, DR_B0086, DR_B0087, respectively) were present in one operon along with a naturally truncated regulatory component *kdpD-N* (representing N-terminal half of the *E*. *coli kdpD*, DR_B0088) (Fig 1). The remaining regulatory components, sensor kinase (*SK*, representing C-terminal half of the *E*. *coli kdpD*, DR_B0082) and the response regulator (*RR*, DR_B0081) were present in a separate operon, in an opposite orientation, immediately upstream of the *kdpBACD-N* operon (Fig 1) [24]. Homologs of such truncated *kdpD* genes are found in the genomes of *Deinococcus-Thermus* group and cyanobacteria (Fig 1), that share close evolutionary relationship [25]. In cyanobacteria, that harbor 2 separate *kdp* operons in yet another distinct organization of regulatory components (Fig 1), only one *kdp* operon responds to potassium limitation, high osmolarity or desiccation [14, 26–28]. However, the role and regulation of *kdp* operon in the normal physiology and stress responses of *D*. *radiodurans* remains completely unexplored.

In this study, we report that the *kdp* operon of *D*. *radiodurans* is induced following K^+^ limitation but does not respond to high osmolarity, unlike *E*. *coli* Kdp ATPase. We also demonstrate that regulation of *kdp* operon is effected through the cognate response regulator (RR) protein. The knockout mutant of RR (ΔRR) displayed *kdp* expression null phenotype even under potassium limiting conditions. In rich media, the growth of ΔRR mutant was similar to wild type *D*. *radiodurans*. Survival response of ΔRR mutant to other relevant stresses was also evaluated.

## Materials and methods

### Bacterial strains and growth conditions

All the strains used in this study are listed in Table 1. Wild type *D*. *radiodurans* strain R1 ATCC BAA-816 or mutant constructed therein, were grown under standard growth conditions [32°C with 150 rpm agitation]. The cells were cultured either in TGY (1% Bacto Tryptone, 0.5% Bacto Yeast Extract, 0.1% glucose) broth, or in minimal medium adapted from previous studies [30–32] with modifications. The composition of the minimal medium was: 20mM Na/K phosphate buffer, 0.5% glucose, 0.05% casamino acids, macronutrients: 2.5mM (NH_4_)_2_SO_4_, 0.1mM CaCl_2_, 5μM FeSO_4_, 0.5mM MgCl_2_, micronutrients: 2nM CuSO_4_, 50nM MnCl_2_, 10nM ZnSO_4_, 50nM CoCl_2_, nicotinic acid (1μg/ml), biotin (1μg/ml). Liquid or solid (1.5% Bacto agar) media were supplemented with 10 μg/ml kanamycin when required. The plasmid constructs were maintained or propagated in *E*. *coli* JM109 while *E*. *coli* BL21(DE3)pLysS strain was used for the over-expression of recombinant proteins (Table 1). All *E*. *coli* strains were grown at 37°C either in LB (Luria-Bertani) broth with agitation (150 rpm), or on LB agar plates supplemented with 50μg/ml kanamycin or 100μg/ml carbenicillin, when necessary. Alternatively, *E*. *coli* BL21(DE3)pLysS cells harboring pET29b vector with desired gene were grown in auto-induction medium [33] at 20°C with 150 rpm agitation.

**Table 1:** Bacterial strains, plasmids and primers used in this study.

**Bacterial strains**		
**Strain**	**Description**	**Reference/ Source**
***Deinococcus radiodurans***		
R1	Wild type strain ATCC BAA-816	[15]
ΔRR	*ΔDR_B0081 D*. *radiodurans* R1 strain, Kan^r^	This study
***E*. *coli***		
JM109	F′traD36 proAB^+^ lacI^q^ lacZΔM15/Δ(lac-proAB) glnV44 e14- gyrA96 recA1 relA1 endA1 thi-1 hsdR17 mcrB^+^	New England Biolabs
JM110	F′traD36 lacI^q^ lacZΔM15 proAB^+^ rpsL thr leu thi lacY galK galT ara tonA tsx dam dcm glnV44 Δ(lac-proAB)	New England Biolabs
*E*. *coli* BL21 (DE3) pLysS	F^– ^ompT gal dcm lon hsdS_B_(r_B_^- ^m_B_^-^) λ(DE3 [lacI lacUV5-T7 gene 1 ind1 sam7 nin5]), Kan^r^.	Novagen
**Plasmids**		
**Plasmids**	**Description**	**Reference**
pUC4K	3.91 kb, Kan^r^	Amersham
pBlueScript	2.96 kb phagemid cloning vector; Amp^r^	Stratagene
pET29b	5.4 kb, pBR322 origin, Kan^r^	Novagen
pET-kdpB	7.4 kb, *NdeI/XhoI* fragment of *DR_B0083* ORF (2028 bp) cloned in *NdeI/XhoI* restricted pET29b, Kan^r^	
pET-skdpB	6.3 kb, *NdeI/XhoI* fragment of soluble portion of *DR_B0083* ORF (914 bp) cloned in *NdeI/XhoI* restricted pET29b, Kan^r^	This study
pET-RR	6.0 kb, *NdeI/XhoI* fragment of *DR_B0081* ORF (630 bp) cloned in *NdeI/XhoI* restricted pET29b, Kan^r^	This study
pΔRR1	4.08 kb, pBS carrying 0.54 kb of sequence flanking the 5′ and 3′ ends of *DR_B0081* (DR_B0081-up and DR_B0081-dn); for the purpose of deleting the chromosomal copy of *DR_B0081*, Amp^r^.	This study
pΔRR2	5.33 kb, pΔ*RR*1 with Kan^r^ cassette inserted between DR_B0081-up and DR_B0081-dn, Amp^r^, Kan^r^.	This study
**Primers**		
**Primer**	**Sequence**	**Reference**
**Primers for cloning *DR_B0083* (kdpB) and *DR_B0081* (RR) genes**		
kdpB-F	5’- TTTCCGGGTACCCATATGACCACGGCCCCTCAG—3’ (KpnI, NdeI)	This study
kdpB-R	5'- AACGCCCTCGAGTGACATCAATCCACC -3' (XhoI)	This study
skdpB-F	5'-TATAACATATGGATAGGGCTTTGCAG-3' (NdeI)	This study
skdpB-R	5'-TCGTTCTCGAGGGAAAAAGTGGTCAGGGC-3'(XhoI)	This study
RR-F	5’-TAAACGGTACCCATATGCCTGACCCGGTGGGC-3’ (KpnI, NdeI)	This study
RR-R	5’-ATATACTCGAGCAGCAGCCCCGCCCG-3’ (XhoI)	This study
**Primers for *DR_B0081* (RR) knockout mutagenesis**		
RR-up-F	5’-ACACAGGTACCAGCTGAACTACGTAGCGA-3’ (KpnI)	This study
RR-up-R	5’-ACACAGATATCTGGCCTTTTGTTGTGGAT-3’ (EcoRV)	This study
RR-dn-F	5’-AGATAGATATCAGCAGGATGCCCACCGGG-3’ (EcoRV)	This study
RR-dn-R	5’-AGATAGGATCCGCCAACGAAACGCTGCTC-3’ (BamHI)	This study
**Primers for promoter cloning**		
P200bp-F	5'-AATATAAGCTTCGGCGGCGGGAACAGCTC-3' (HindIII)	This study
P200bp-R	5'-AATATGATATCCCTGGTCGCGCGGCGAGA-3' (EcoRV)	This study
P38-F	5'-AACGGCGCTATGAGTCTTTCTTCTTTCCGGTGGCGGCC-3’	This study
P38-R	5'-GGCCGCCACCGGAAAGAAGAAAGACTCATAGCGCCGTT-3’	This study
**Primers for sequencing**		
kdpB-548-F_Seq	5'- CATCGTCATTCAAATCACCTC -3'	This study
kdpB-1098-F_Seq	5'- GAGTTCATCGAATTCACCGCC -3'	This study
kdpB-1620-F_Seq	5'- AACATGGTGGATTTGGACAGC -3'	This study
**Primers for non-specific DNA**		
0906RTF	5’-TTTATCCACGCCAACACCTA-3’	This study
0906RTR	5’-GGCCTTGATGAGGTTCTTGT-3’	This study

### Stress conditions

*D*. *radiodurans* cells or its mutant was grown overnight in TGY medium. The cells were washed twice in either TGY, 20 mM K^+^ phosphate supplemented minimal medium (hereafter referred to as K20 medium) or 20 mM sodium phosphate supplemented minimal medium (hereafter referred to as K0 medium) and the washed cells were inoculated in TGY, K20 medium or K0 medium, respectively, at an initial cell density of OD_600_ = 0.5/ml. The cultures were incubated at 32°C with 150 rpm agitation. The cells resuspended in K0 medium experienced K^+^ limitation while the cells resuspended in K20 medium or TGY served as controls. For ionic or osmotic stress, K1 medium (1:20 dilution of K20 medium in K0 medium) was supplemented with either 0.1M NaCl or 0.2M sucrose. For gamma irradiation experiments, overnight grown cells of wild type or mutant *D*. *radiodurans* cells were resuspended in fresh TGY medium (OD_600_ = 3.0), 2 μl aliquots of the cell suspensions and their serial dilutions were spotted onto the TGY plate. The plate was exposed to 5 kGy gamma irradiation (Gamma Cell 5000, Bhabha Atomic Research Centre, dose rate: 1.85 kGy/hr). Following stress, the plate was incubated at 32° C for 24 h for recovery before the result was scored. For heat and oxidative stresses, the overnight grown cells of wild type or mutant *D*. *radiodurans* cells were resuspended in fresh TGY medium (OD_600_ = 0.5) and exposed to either 42° C or 100 mM H_2_O_2_ for 1h with 150 RPM agitation. Further, the cells were concentrated (OD_600_ = 3.0) and 2 μl aliquots of the cell suspensions and their serial dilutions were spotted onto the TGY plate. The plate was incubated at 32° C for 24 h for recovery before the result was scored.

### Cloning of *DR_B0083* (complete or partial) and *DR_B0081* ORFs, and over-expression and purification of corresponding proteins

Genomic DNA of *D*. *radiodurans* was prepared as per the protocol reported previously [34]. The *DR_B0083* (*kdpB*), *DR_B0083/826-1740bp* (s*kdpB*), *DR_B0081* (RR) ORFs were PCR amplified from the genomic DNA of *D*. *radiodurans* using gene-specific primers listed in Table 1. The PCR products were restriction digested using *NdeI/XhoI* and cloned into pET-29b plasmid vector at the identical sites. Correct clone was ascertained by DNA sequencing. The resultant constructs pET-kdpB, pET-skdpB or pET-RR were transformed in *E*. *coli* BL21(DE3)pLysS and expression of corresponding proteins was induced in auto-induction medium at 20°C. The cells were harvested after 15h of growth and the over-expressed proteins (sKdpB and RR) were purified by affinity chromatography using Ni-nitrilotriacetic acid (Ni^+2^-NTA)-agarose resin (Qiagen, Germany), as per the manufacturer’s protocol. The purified RR protein was used for promoter interaction studies. The sKdpB and RR proteins were purified by gel elution method and used to generate polyclonal antibodies in rabbit, at a commercial facility of Bangalore Genei (India).

### Deletion of *DR_B0081* (RR) gene and confirmation of the knockout mutant

The Δ*DR_B0081* mutant was constructed as per the procedure described previously [35]. In brief, mutagenesis strategy involved complete replacement of *DR_B0081* ORF with an *aph* cassette that conferred kanamycin resistance. For this purpose, the upstream (RR-up) and downstream (RR-dn) sequences (540 bp each) of *DR_B0081* were PCR amplified using primer pairs RR-up-F/R and RR-dn-F/R, respectively (Table 1). The PCR amplified RR-up and RR-dn DNA fragments were restriction digested with KpnI/EcoRV and EcoRV/BamHI, respectively and were ligated to the KpnI/BamHI sites of pBlueSkript vector to obtain plasmid pΔRR1. To obtain *aph* cassette, plasmid pUC4K was restriction digested with HincII to release the 1252 bp DNA fragment harboring *aph* cassette. The *aph* cassette was cloned into the EcoRV site of pΔRR1 plasmid, by blunt end ligation. The resultant construct pΔRR2, with *aph* cassette inserted between the RR-up and RR-dn sequences, was used to transform WT *D*. *radiodurans*. Kanamycin resistant transformants were selected on TGY plates containing 10μg/ml kanamycin. The ΔRR knockout mutant was confirmed by PCR and western blot analysis of RR protein.

### Immuno-detection of KdpB and RR proteins

The cellular extracts were prepared by sonication (Branson Digital Sonifier, Model 250). Cell debris was removed by centrifugation (10000 rpm for 10 min). The supernatant was centrifuged at 100000g for 1h. The supernatant was collected as cytosolic fraction. The pellet was washed 2 times and then collected as membrane fraction. The cellular proteins extracts, cytosolic or membrane fractions from stressed or unstressed cells were resolved by 12% SDS-PAGE and electro-blotted onto nitrocellulose membrane (Amersham Biosciences, India). The presence of KdpB or RR proteins was probed with primary polyclonal anti-*D*. *radiodurans*-sKdpB or anti-*D*. *radiodurans*-RR antibodies (1:10,000 dilution), respectively, followed by goat anti-rabbit IgG tagged to alkaline phosphatase (Sigma, 1:5000 dilution). The western blots were developed using NBT-BCIP solution (Roche, Germany). The immuno-detected protein bands which appeared on the membranes were quantified using CLIQS 1D PRO (Total Labs, UK).

### Electrophoretic mobility shift assay (EMSA)

The promoter region (200 bp, DNA sequence immediately upstream of DR_B0083 i.e. *kdpB* gene) of *kdp* operon was PCR amplified using specific primers (Table 1). Alternatively, the intergenic region (38 bp) between *DR_B0082* (sensor kinase) and *DR_B0083* (*kdpB*) was generated *in vitro* by two primer (P38-F/ P38-R) annealing (Table 1). The DNA fragments were labeled with DIG-ddUTP (Digoxigenin) as per manufacturer’s protocol (Roche, India). DIG labeled promoter fragment (45 ng of DIG-labeled 200 bp DNA) was incubated with indicated concentrations of RR protein in binding buffer [20 mM HEPES pH 7.6,10 mM (NH4)_2_SO_4_, 30 mM KCl, 1 mM EDTA, 1 mM DTT and 0.2% Tween20 (v/v)] at 37°C for 1 h. RR protein was phosphorylated using acetyl phosphate as per the procedure detailed earlier [28]. The DNA–protein complexes were resolved on 10% native polyacrylamide gel at 50 V in 0.5X TBE gel running buffer. The resolved DNA–protein complexes were electro-blotted onto nylon membrane for 30 mins. The nylon membrane was cross-linked with UV, probed with anti-DIG antibody and subsequently developed using colorimetric substrate, NBT-BCIP. Each experiment was repeated three times. The bands which appeared on membranes were quantified using gel quant software (Biochem lab solutions) and the K_D_ value for RR protein was obtained by fitting the data to Hill’s equation [36]. Specificity of RR interaction was confirmed by titration of DNA-protein complexes with unlabeled target-specific or non-specific DNA. Further, to determine specific interaction of RR protein with the *kdp* promoter, EMSA was carried out either by replacing *kdp* promoter fragment with non-specific DNA or by replacing RR with BSA protein. Non-specific DNA was a DNA sequence (153) bp from DR_0906 gene amplified using primers listed in Table 1.

### Bioinformatic analyses

BLASTP [37] and ClustalW [38] tools were used to analyze sequence similarities and for phylogenetic analyses of the proteins encoded by *kdp* operon of *D*. *radiodurans*. Presence of *E*. *coli* like gene promoter elements in the upstream sequence of *kdp* operon was analyzed using BPROM software [39]. Phobius software was used to predict presence of a transmembrane domain in KdpB protein [40].

## Results and discussion

### The *kdp* operon of *D*. *radiodurans* responds to K^+^ limitation

To ascertain if the *kdp* operon of *D*. *radiodurans* responds to K^+^ limitation, we cloned the genes for soluble domain of KdpB (sKdpB, amino acids 277–573 of KdpB protein) and response regulator protein, and over-expressed and purified the corresponding proteins and raised polyclonal antibodies (S1 Fig). The genes for full length *kdpB* and sensor kinase were also cloned but expression of corresponding proteins could not be achieved in *E*. *coli* despite several attempts.

**Fig 2:**
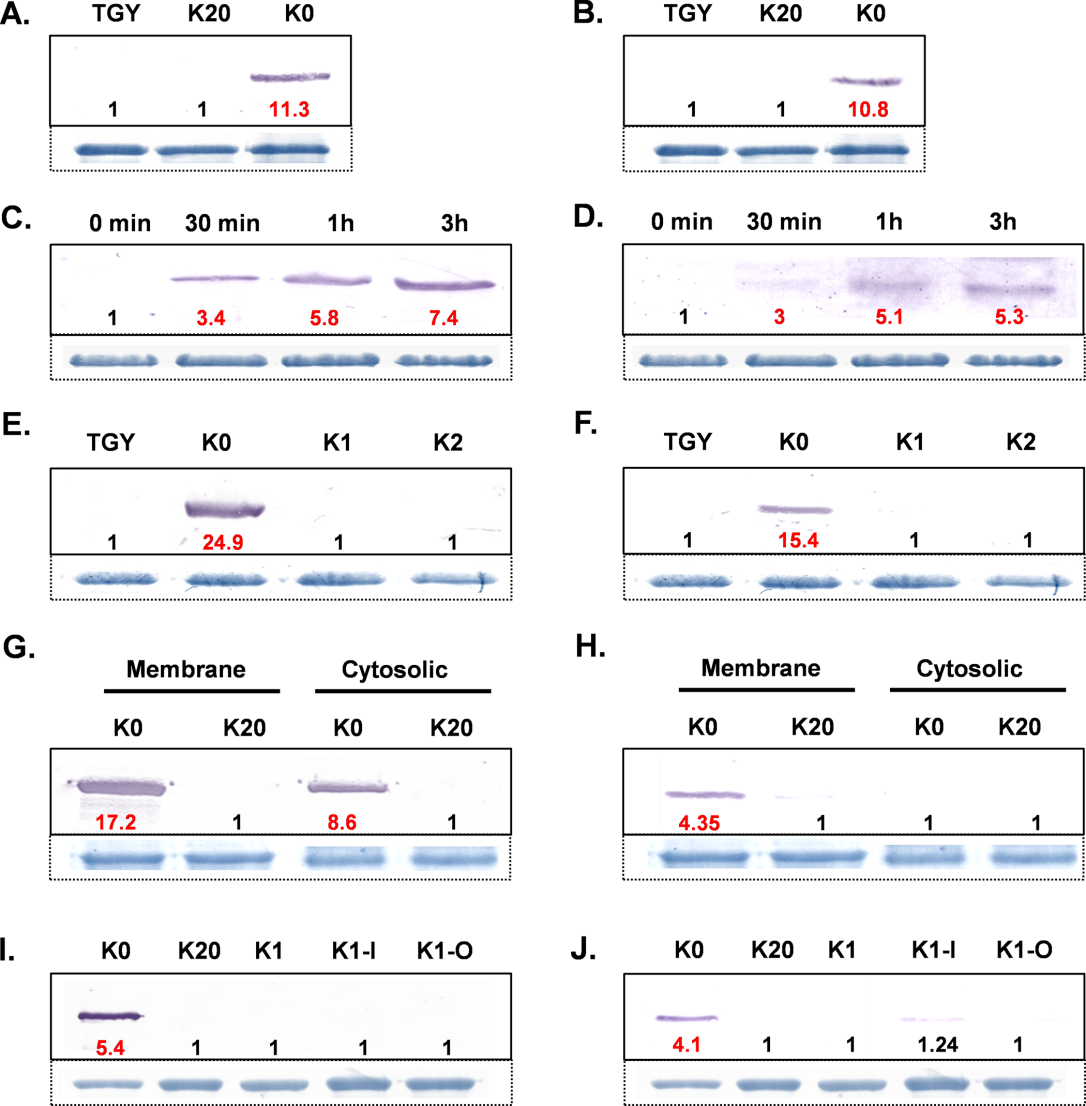
KdpB and RR expression under different growth conditions. Expression of KdpB (A) or RR (B) proteins in *D*. *radiodurans* cells incubated in TGY, K20 or K0 media. Time course of induction of KdpB (C) or RR (D) proteins in *D*. *radiodurans* cells following shift from TGY to K0 medium. Expression of KdpB (E) or RR (F) proteins in *D*. *radiodurans* cells incubated in TGY, K0, K1 or K2 media. Localization of KdpB (G) or RR (H) proteins in *D*. *radiodurans* cells incubated in K20 or K0 media. Expression of KdpB (I) or RR (J) proteins in *D*. *radiodurans* cells grown either in K20 or K0 media, or exposed to ionic (-I, 0.1M NaCl) or osmotic (-O, 0.2M sucrose) stresses in K1 medium. The cellular proteins (100 μg) were resolved by 12% SDS-PAGE, electroblotted onto nitrocellulose membrane and immuno-stained using anti-KdpB or anti-RR antibodies as detailed in materials and methods. The top most protein band (~ 125 kDa) in the corresponding Coomassie stained gel is shown below Fig 2A, 2B, 2C, 2D, 2E, 2F, 2I and 2J, as loading control. For membrane or cytosolic protein extracts, 63 kDa protein band or 44 kDa protein band, respectively, are shown as loading controls (See S2 Fig for details on loading controls). Red bold numbers below the KdpB or RR immuno-stained bands indicate fold increase in their levels over the lanes in which these bands were not observed (denoted by 1).

We probed the induction of KdpB as well as RR proteins in response to K^+^ limitation. The KdpB as well as RR proteins were undetectable in *D*. *radiodurans* cells grown in TGY medium or K20 medium, but the cells incubated in K0 medium showed prominent induction of both KdpB and RR proteins (Fig 2A and 2B). The result shows that the *kdp* operon of *D*. *radiodurans* responds to K^+^ limitation. The induction of RR protein post K^+^ limitation is intriguing since in *Anabaena* L-31, the basal level of RR remains unaffected by K^+^ limitation [28], while in *E*. *coli* as well as in *Clostridium acetobutylicum* the expression of KdpE increases as a result of synthesis of a polycistronic mRNA encompassing *kdpABCDE* genes and a read-through effect [41, 42]. Further, we checked the kinetics of gene expression and observed that both the KdpB and RR proteins were synthesized within 30 minutes of transfer of *D*. *radiodurans* cells from TGY medium to K0 medium (Fig 2C and 2D). The response to K^+^ limitation was further assessed at different concentrations of K^+^. Both KdpB as well as RR proteins were induced in K0 medium but not in K1 medium (1 mM K^+^ phosphate added to K0 medium) (Fig 2E and 2F), indicating that unlike *E*. *coli*, the *kdp* operon of *D*. *radiodurans* could be induced only below 1 mM K^+^ concentration. Similar results were observed for *Anabaena* L-31 earlier [28]. While most of the KdpB protein localized in membrane (Fig 2G), the RR protein was entirely localized in the membranes (Fig 2H). RR is expected to be a cytosolic protein as it does not possess membrane anchoring or spanning domains. RR protein, when over-expressed in heterologous *E*. *coli*, localized entirely in the cytoplasm. In *D*. *radiodurans*, DNA is present in the nucleoid [43] and about 70% of the DNA is associated with the membranes [44]. The localization of RR protein in the membrane may be due to its possible interaction with *kdp* operon signaling proteins (KdpD-N and SK) localized in the membrane and its functionality can be facilitated by association of DNA with the membranes.

### The *kdp* operon of *D*. *radiodurans* does not respond to ionic or osmotic stresses

In addition to response to K^+^ limitation, the *kdp* operon is also induced by high osmolarity in bacteria [45–47]. In *E*. *coli* K12, the expression of *kdp* operon was about 10-fold higher in response to K^+^ limitation as compared to its expression in response to increased osmolarity by 0.4M NaCl, which in turn is about 10-fold higher compared to its expression in response to 0.6M sucrose stress [48]. Expression of *kdp* operon was originally proposed to be in response to changes in turgor pressure, but was but later debated [49–51]. Here we show that the *kdp* operon of *D*. *radiodurans* does respond to K^+^ limitation but does not respond at all to increased osmolarity. We changed the osmolarity of the K1 medium either by adding 0.1M NaCl or 0.2M sucrose and checked induction of KdpB and RR proteins. Neither KdpB nor RR protein were induced upon addition of NaCl or sucrose (Fig 2I and 2J). The *kdp* operon of *Anabaena* L-31, signaling components of which are similar to those of *kdp* operon of *D*. *radiodurans*, does respond to NaCl stress but does not respond to sucrose [27], although, *kdp* operon of another cyanobacterium *Anabaena torulosa* does respond to sucrose [26]. In contrast, the *kdp* operon of *D*. *radiodurans* appears to be highly specific for K^+^ limitation alone, and free from the influences of osmolarity.

### RR protein induces *kdp* operon under K^+^ limitation

**Fig 3:**
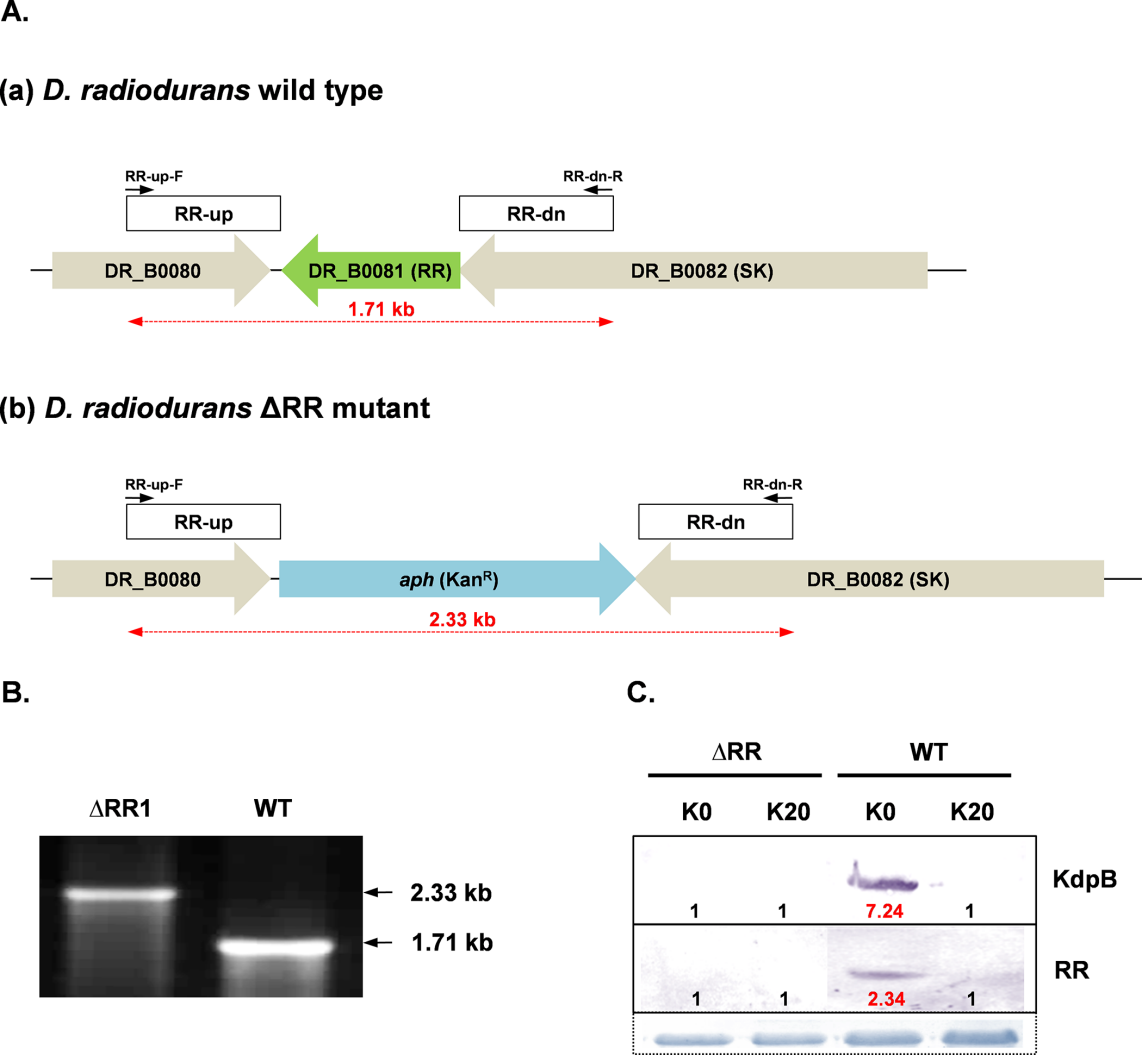
Construction and confirmation of ΔRR mutant. (A) Schematic representation of the RR gene (DR_B0081) in wild type *D*. *radiodurans* (a) and its replacement with kanamycin resistance cassette (*aph*) in ΔRR *D*. *radiodurans* (b). The primers used for the PCR confirmation of the mutant are shown. (B) Confirmation of complete deletion of RR gene in ΔRR *D*. *radiodurans* as compared to wild type *D*. *radiodurans*, using primer pair shown in Fig 3A. (C) Expression of KdpB or RR proteins in wild type or ΔRR *D*. *radiodurans* cells incubated in K0 or K20 media. Details of immuno-staining, loading controls and fold change levels were same as described in legend to Fig 2.

Comparison of the RR protein encoded by *DR_B0081* gene of *D*. *radiodurans* revealed 23.9% identity with KdpE of *E*. *coli*. BLAST analysis also revealed homology of RR protein with similar two-component response regulators from *Deinococcus-Thermus* group and cyanobacteria. Therefore, it was pertinent to ascertain if the RR protein was indeed the response regulator of the *kdp* operon of *D*. *radiodurans*. We constructed a knockout mutant of RR by replacing the *DR_B0081* gene with *aph* gene conferring kanamycin resistance, by site directed mutagenesis, as per the strategy outlined in Fig 3A. The knockout mutant was confirmed by PCR analysis. When RR-up-F/RR-dn-R primer pair was used, 2.33 kb and 1.71 kb bands were amplified from the genomic DNA of the prospective RR knockout mutant and wild type *D*. *radiodurans*, respectively, thereby confirming that the 0.63 kb *DR_B0081* gene had been replaced by 1.25 kb *aph* cassette (Fig 3A and 3B). The mutant thus obtained failed to induce KdpB upon K^+^ limitation. KdpB as well as RR proteins were induced in wild type *D*. *radiodurans* under similar culture conditions (Fig 3C). The data confirmed that the RR protein encoded by DR_B0081 gene was indeed responsible for expression of Deinococcal *kdp* operon under potassium limitation.

### RR protein binds to an intergenic DNA sequence between structural and signaling genes of *kdp* operon

**Fig 4:**
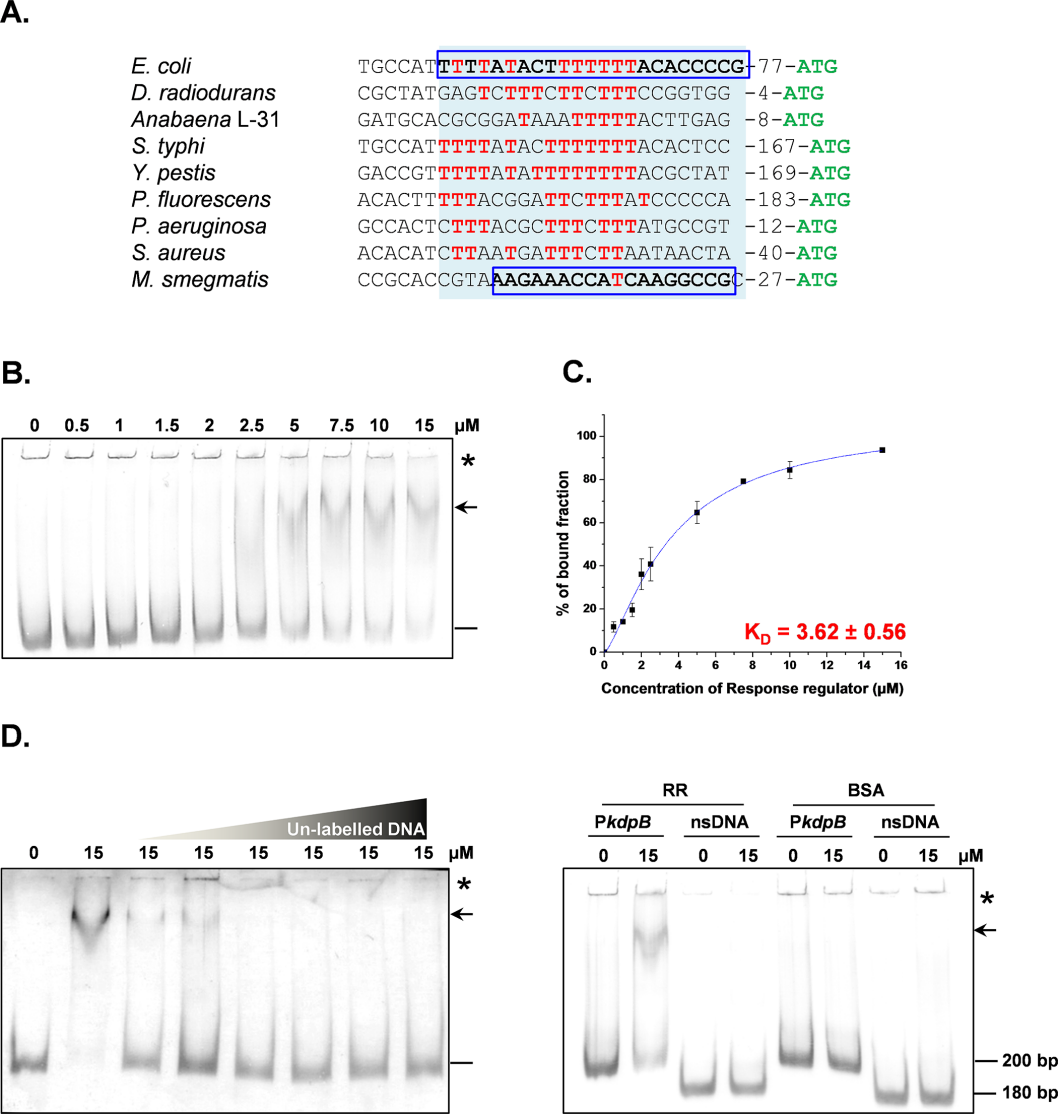
[T_n_]-rich region present in the *kdp* promoter region in various bacteria. (A) [T_n_]-rich region is shown in pale blue box. The number after the [T_n_]-rich region indicate the number of bases between the [T_n_]-rich region and the start codon. [T_n_]-rich and [A_n_]-rich *Kdp*E binding site of *E*. *coli* and *M*. *smegmatis*, respectively, are shown in blue boxes. [T_n_]-rich sequences in the upstream regions of *kdpB* gene in other bacteria are shown in red bold letters. (B) Binding of RR protein to the P*kdpB*-200 of *D*. *radiodurans*. The indicted concentrations of RR protein were incubated with P*kdpB*-200 promoter (45 ng of DIG-labeled 200 bp dsDNA) at 37°C for 1 h and the DNA–protein complexes were resolved by 10% native PAGE. The amount of DNA-protein complexes were estimated using GelQuant software. Substrate DNA and DNA-RR complex are shown by “—” and “←”, respectively, while wells of the gels are marked by asterisk. (C) The representative graph for DNA protein complexes. The data-points were fitted into Hill’s equation (dotted line) to determine K_D_ value. (D) Titration of RR-promoter complexes with unlabeled promoter DNA. (E) Interaction of RR or non-specific protein BSA with P*kdpB*-200 (specific target) or non-specific DNA sequence. For (D) and (E), the DNA-protein complexes were resolved as described in legend to Fig 4B.

[T_n_]-rich region present in the *kdp* promoter region in *E*. *coli* has been shown to bind response regulator KdpE [52, 53]. A similar [T_n_]-rich region is present in the intergenic region (38 bp) between the *kdpB* and *SK* genes of *D*. *radiodurans* and in the upstream sequences of *kdp* operons in other bacteria as well [53, 54] (Fig 4A). Thus, the 38 bp intergenic region harbouring [T_n_]-rich sequence is a potential site for regulation of *kdp* operon. This short sequence did not possess any sequence elements essential for gene expression as analysed by BPROM software. However, several of the Deinococcal promoters are devoid of the typical *E*. *coli* type sequence elements but are still expressed constitutively [55]. Here, we chose a short (38 bp) as well as longer sequence (200 bp) for our study. We determined the binding of RR protein to the promoter sequence of *kdpBACD* operon (P*kdpB*-38/ P*kdpB*-200). Retardation in the mobility of P*kdpB*-200 incubated with increasing concentrations of RR protein confirmed binding of RR to P*kdpB*-200 (Fig 4B). We obtained a similar profile of retardation in the mobility of P*kdpB*-200 incubated with phosphorylated RR protein (S3 Fig). The apparent equilibrium dissociation constant (K_D_) was calculated to be 3.62 ± 0.56 μM, which signifies the amount of protein required for 50% binding to P*kdpB*-200. The Hill coefficient value was determined to be 1.36 ± 0.17. Thus, RR showed very low or no co-operativity in binding (Fig 4C). Similarly, the mobility of P*kdpB*-38 was also retarded in the presence of increasing concentrations of RR protein (S4 Fig), confirming that this small intergenic region indeed provided the site for RR binding. To probe specific interaction of RR protein with P*kdpB*-200, competitive EMSA was carried out by titrating the DIG-labelled P*kdpB*-RR complexes with unlabeled P*kdpB*-200. With increasing concentration of unlabelled P*kdpB*-200, the labelled P*kdpB*-200 DNA was released from the P*kdpB*-200-RR complexes, where the labelled free DNA was clearly visible following competition with increasing amounts of unlabeled specific DNA (Fig 4D). Non-specific interactions of RR with nsDNA or of P*kdpB*-200 with any protein were ruled out by either incubating RR protein with P*kdpB*-200 or with nsDNA, or incubating BSA with P*kdpB*-200 or with nsDNA. DNA-protein complexes were observed only when P*kdpB*-200 was incubated with RR (Fig 4E), thereby confirming the specific interaction of RR with P*kdpB*-200.

### Relevance of *kdp* operon to stress resistance of *D*. *radiodurans*

**Fig 5:**
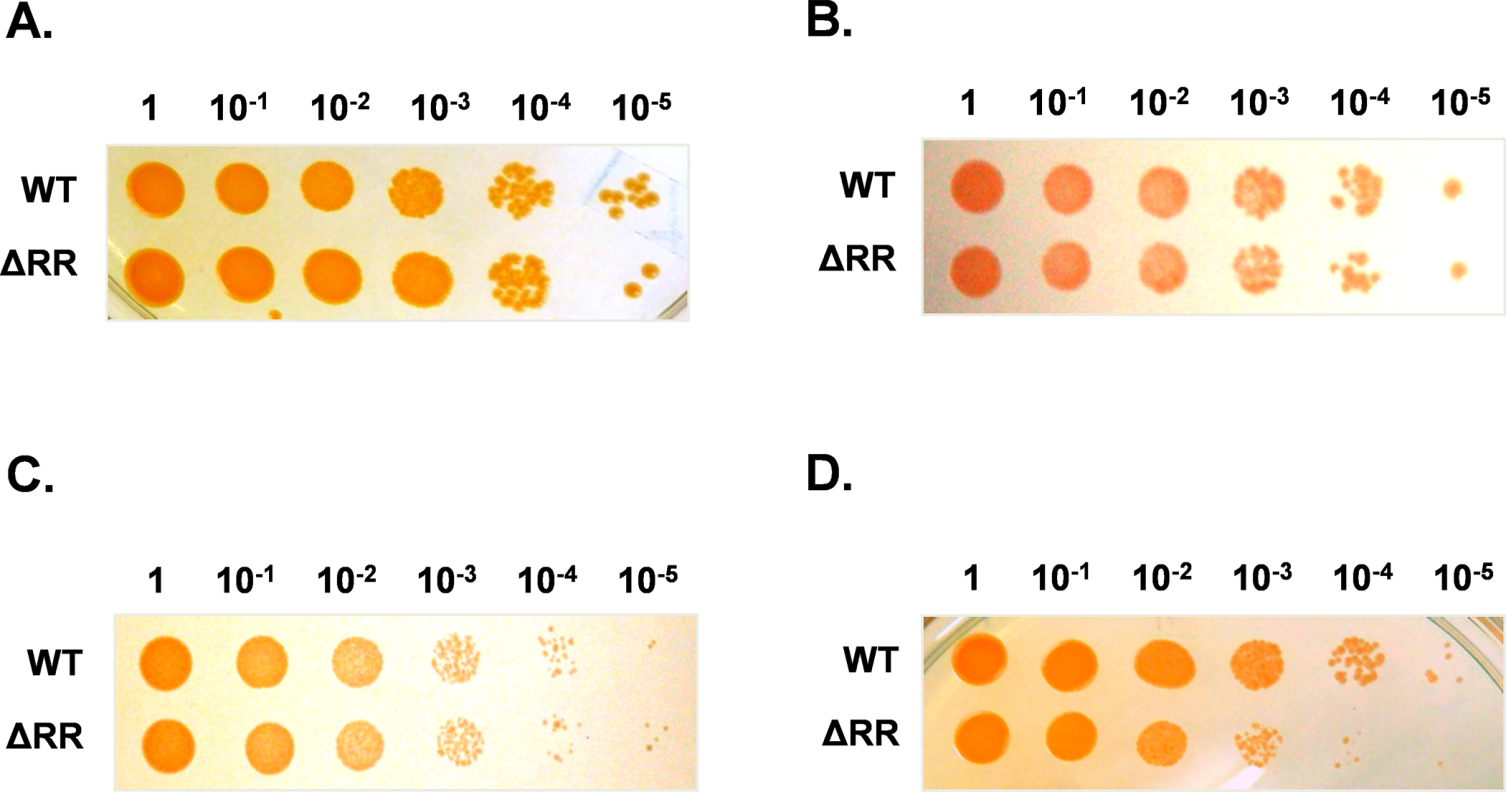
**Survival of wild type and ΔRR mutant of *D*. *radiodurans*** (A) under standard growth conditions, (B) exposed to 100 mM H_2_O_2_, (C) exposed to 42° C or (D) exposed to 5 kGy gamma irradiation.

Since *kdp* operon is reported to give survival advantage under stressful conditions in both Gram-positive as well as Gram-negative bacteria [4–12], we checked if presence of inducible *kdp* operon offered any advantage to *D*. *radiodurans* during stress. We used a *kdp* expression null ΔRR mutant to evaluate survival fitness following different stresses. The ΔRR mutant did not show any growth defect, as compared to wild type *D*. *radiodurans*, when grown in TGY medium under standard growth conditions (Fig 5A), or when exposed to 100 mM H_2_O_2_ (Fig 5B) or 42° C heat stress (Fig 5C). However, survival of ΔRR mutant was reduced, as compared to wild type *D*. *radiodurans*, following 5 kGy gamma irradiation (Fig 5D).

The study, for the first time, reports functional characterization of the *kdp* operon of the radioresistant extremophile *D*. *radiodurans*, having a distinct operon organization. Unique features of the *kdp* operon of *D*. *radiodurans* include (a) induction of both, the structural and regulatory components, of the *kdp* operon under extreme potassium limitation, (b) lack of stimulation by increased osmolarity either by NaCl or sucrose, (c) expression of *kdp* operon through specific interaction of unphosphorylated RR protein with *kdp* promoter, and (d) confers a minor survival advantage to the organism while recovering from gamma radiation stress.

## Supporting information

S1 Fig Over-expression, purification of sKdpB and RR proteins. The gel image shows sKdpB and RR proteins were overexpressed in *E*. *coli* BL21(DE3)pLysS (30 μg protein/lane) and purified sKdpB and RR proteins (5 μg protein/lane) in lanes 1–2 and 3–4, respectively. Molecular weight marker (SDS-7, Sigma) are shown in lane M. The purified RR protein was used for promoter interaction studies. The sKdpB and RR protein bands (shown by arrows on right hand side) were purified by gel elution method and were further used for generation of polyclonal antibodies in rabbit.
(TIF)

S2 Fig A representative gel image showing loading controls. Whole cell protein extract (Lane 1), cytosolic fraction (Lane 2) and membrane fraction (Lane 3) were resolved by 12% SDS-PAGE. Molecular weight markers (P7712L, NEB) are shown in lane M. The 125 kDa (S-layer protein, DR_2577, *), 63 kDa (ABC transporter-binding protein, DR_1571, **) and 44 kDa (Elongation factor Tu, DR_0309, ***) were used as loading controls for whole cell protein extract, membrane fraction and cytosolic fraction, respectively. For details on the protein identities, please see reference No. 16.
(TIF)

S3 Fig Binding of phosphorylated RR protein to the P*kdpB*-200 of *D*. *radiodurans*. The indicted concentrations of phosphorylated RR protein were incubated with P*kdpB*-200 promoter (45 ng of DIG-labeled 200 bp dsDNA) at 37°C for 1 h and the DNA–protein complexes were resolved by 10% native PAGE. Substrate DNA and DNA-RR complexes are shown by “—” and “←”, respectively, while wells of the gels are marked by asterisk.
(TIF)

S4 Fig Binding of RR protein to the P*kdpB*-38 of *D*. *radiodurans*. The indicted concentrations of RR protein were incubated with P*kdpB*-38 promoter (45 ng of DIG-labeled 38 bp dsDNA) at 37°C for 1 h and the DNA–protein complexes were resolved by 12% native PAGE. Substrate DNA and DNA-RR complexes are shown by “—” and “←”, respectively, while wells of the gels are marked by asterisk.
(TIF)
