# Supplementary figures and images for: Regulation of potassium dependent ATPase (kdp) operon of Deinococcus radiodurans
Source: PLoS One. 2017 Dec 5;12(12):e0188998. doi: 10.1371/journal.pone.0188998 (PMC5716572; doi:10.1371/journal.pone.0188998)

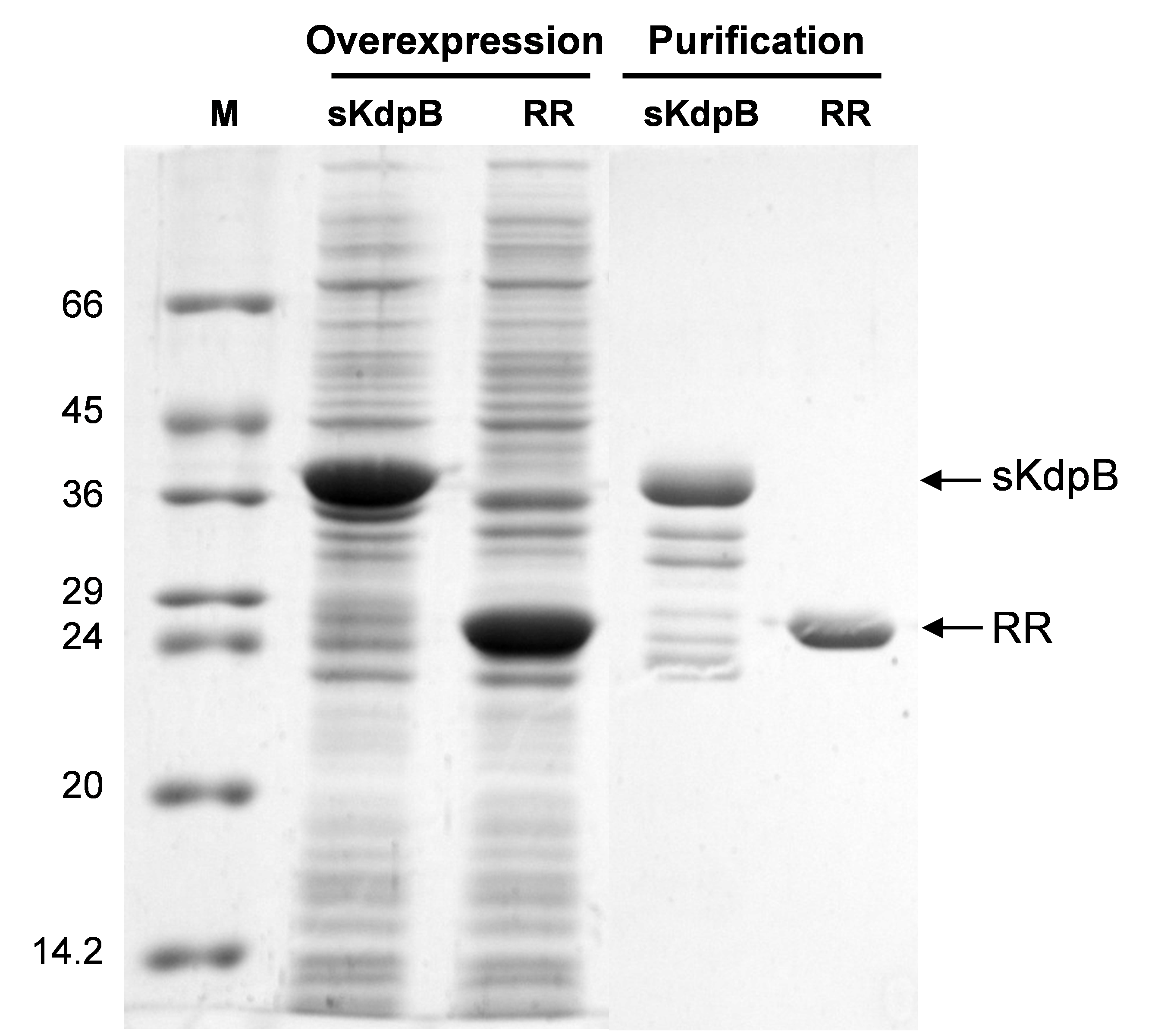

Supplement: S1 Fig — The gel image shows sKdpB and RR proteins were overexpressed in E. coli BL21(DE3)pLysS (30 μg protein/lane) and purified sKdpB and RR proteins (5 μg protein/lane) in lanes 1–2 and 3–4, respectively. Molecular weight marker (SDS-7, Sigma) are shown in lane M. The purified RR protein was used for promoter interaction studies. The sKdpB and RR protein bands (shown by arrows on right hand side) were purified by gel elution method and were further used for generation of polyclonal antibodies in rabbit. (TIF) [file pone.0188998.s001.tif]

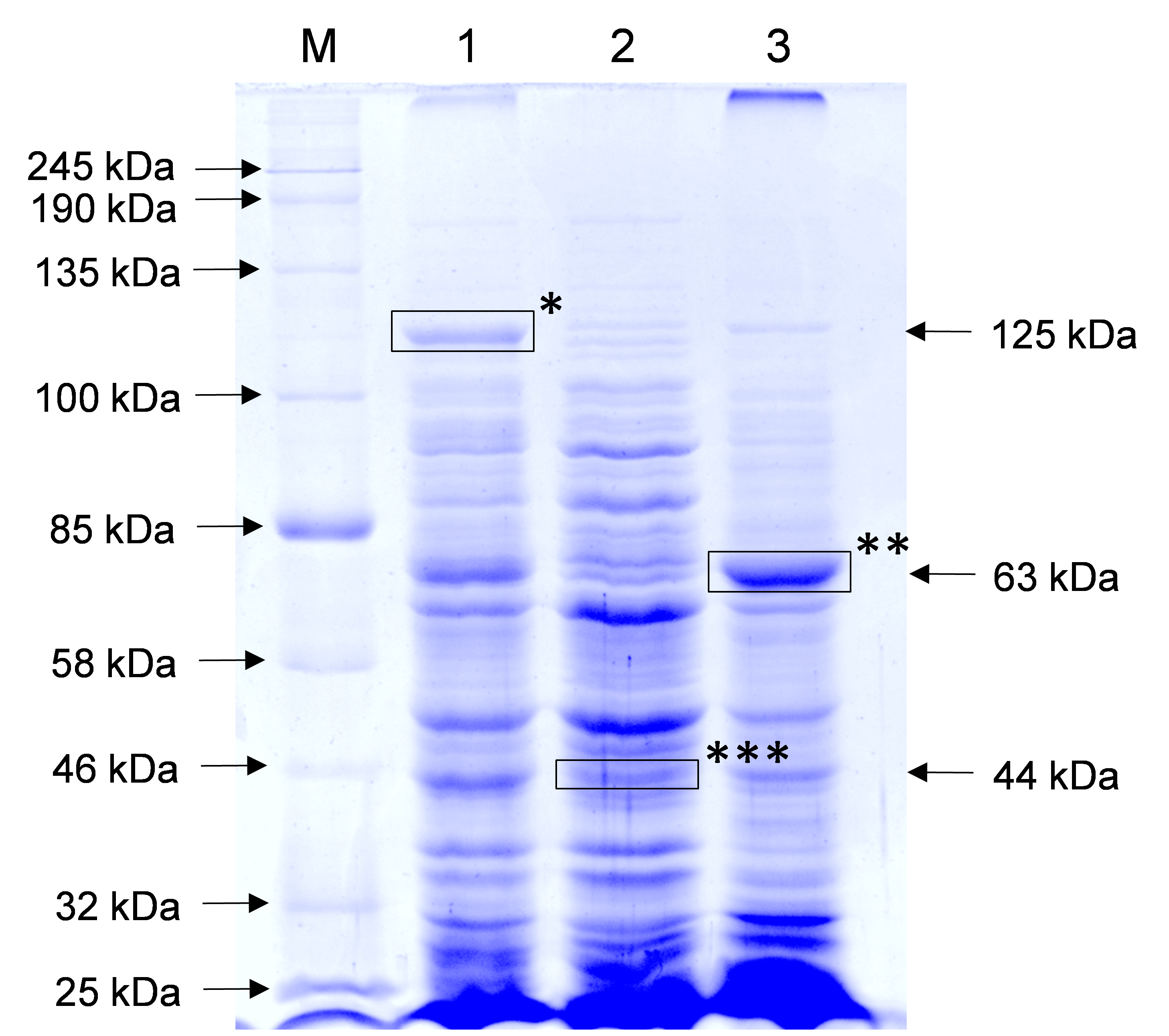

Supplement: S2 Fig — Whole cell protein extract (Lane 1), cytosolic fraction (Lane 2) and membrane fraction (Lane 3) were resolved by 12% SDS-PAGE. Molecular weight markers (P7712L, NEB) are shown in lane M. The 125 kDa (S-layer protein, DR_2577, *), 63 kDa (ABC transporter-binding protein, DR_1571, **) and 44 kDa (Elongation factor Tu, DR_0309, ***) were used as loading controls for whole cell protein extract, membrane fraction and cytosolic fraction, respectively. For details on the protein identities, please see reference No. 16. (TIF) [file pone.0188998.s002.tif]

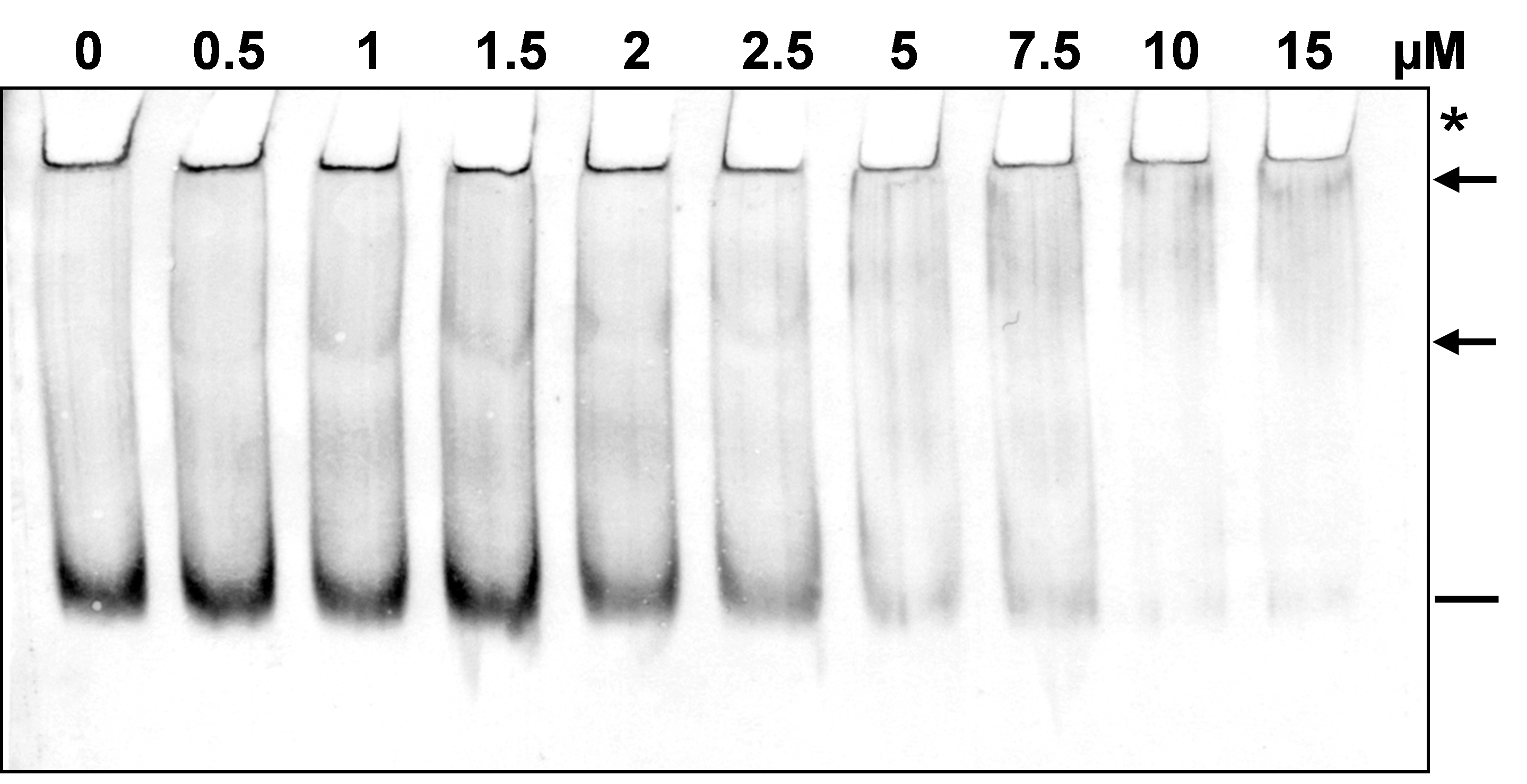

Supplement: S3 Fig — The indicted concentrations of phosphorylated RR protein were incubated with PkdpB-200 promoter (45 ng of DIG-labeled 200 bp dsDNA) at 37°C for 1 h and the DNA–protein complexes were resolved by 10% native PAGE. Substrate DNA and DNA-RR complexes are shown by “—” and “←”, respectively, while wells of the gels are marked by asterisk. (TIF) [file pone.0188998.s003.tif]

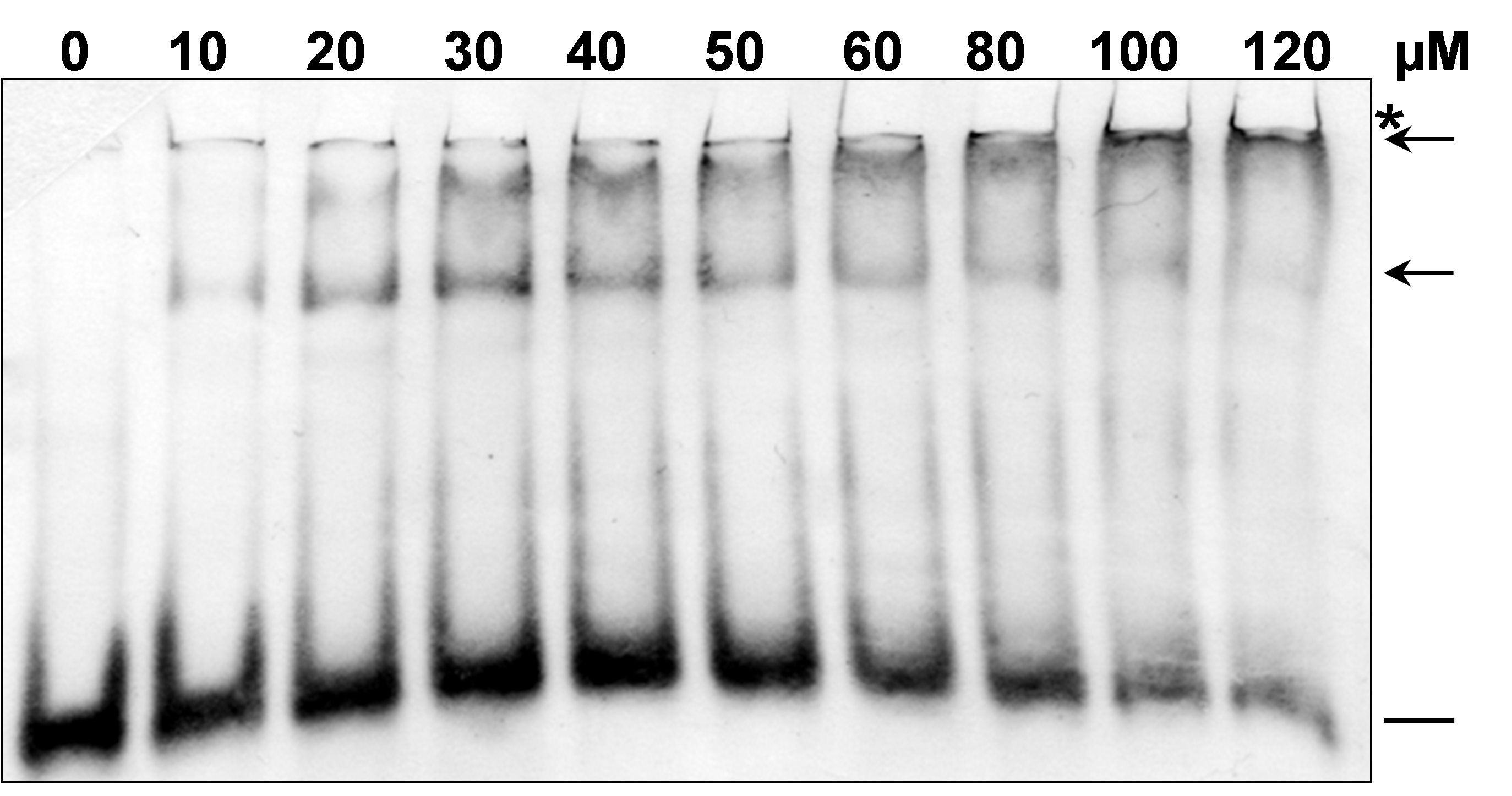

Supplement: S4 Fig — The indicted concentrations of RR protein were incubated with PkdpB-38 promoter (45 ng of DIG-labeled 38 bp dsDNA) at 37°C for 1 h and the DNA–protein complexes were resolved by 12% native PAGE. Substrate DNA and DNA-RR complexes are shown by “—” and “←”, respectively, while wells of the gels are marked by asterisk. (TIF) [file pone.0188998.s004.tif]
